# Increased co-contraction reaction during a surface perturbation is associated with unsuccessful postural control among older adults

**DOI:** 10.1186/s12877-022-03123-2

**Published:** 2022-05-19

**Authors:** Jimmy Falk, Viktor Strandkvist, Mascha Pauelsen, Irene Vikman, Lars Nyberg, Ulrik Röijezon

**Affiliations:** Department of Health, Education and Technology, Luleå University of Technology, Luleå, Sweden

**Keywords:** Ageing, Balance, Co-contraction index, Electromyography, Falls, Sensorimotor, Stiffening strategy, Surface perturbation

## Abstract

**Background:**

As a strategy to maintain postural control, the stiffening strategy (agonist-antagonist co-contractions) is often considered dysfunctional and associated with poor physical capacity. The aim was to investigate whether increased stiffening is associated with unsuccessful postural control during an unpredictable surface perturbation, and which sensory and motor variables that explain postural stiffening.

**Methods:**

A sample of 34 older adults, 75.8 ± 3.8 years, was subjected to an unpredicted surface perturbation with the postural task to keep a feet-in-place strategy. The participants also completed a thorough sensory- and motor test protocol. During the surface perturbation, electromyography was measured from tibialis anterior and gastrocnemius to further calculate a co-contraction index during the feed-forward and feedback period. A binary logistic regression was done with the nominal variable, if the participant succeeded in the postural task or not, set as dependent variable and the co-contraction indexes set as independent variables. Further, the variables from the sensory and motor testing were set as independent variables in two separate Orthogonal Projections of Latent Structures (OPLS)-models, one with the feed-forward- and the other with the feedback co-contraction index as dependent variable.

**Results:**

Higher levels of ankle joint stiffening during the feedback, but not the feed-forward period was associated with postural task failure. Feedback stiffening was explained by having slow non-postural reaction times, poor leg muscle strength and being female whereas feed-forward stiffening was not explained by sensory and motor variables.

**Conclusions:**

When subjected to an unpredicted surface perturbation, individuals with higher feedback stiffening had poorer postural control outcome, which was explained by poorer physical capacity. The level of feed-forward stiffening prior the perturbation was not associated with postural control outcome nor the investigated sensory and motor variables. The intricate causal relationships between physical capacity, stiffening and postural task success remains subject for future research.

**Supplementary Information:**

The online version contains supplementary material available at 10.1186/s12877-022-03123-2.

## Background

In everyday life we face different postural challenges dependent on the environment and the tasks we perform [1]. To successfully maneuver in various settings, we rely on our postural control system that processes sensory input, especially from visual, somatosensory and vestibular systems to further execute appropriate motor actions by our muscles [2]. This is achieved by a constant interchangeable collaboration of anticipatory preparations based on previous experiences i.e., feed-forward actions, and reacting to sensory information i.e., feedback reactions [1]. These actions and reactions have an interdependent relationship; if the feed-forward actions are inadequate, for example during unexpected postural events, greater feedback responses might be necessary to avoid task failure, such as a fall [3, 4]. Different circumstances require different postural strategies to avoid loss of balance and potential falls. For small unexpected postural challenges, it might be sufficient to stiffen the ankle joint by agonist-antagonist co-contractions i.e., the stiffening strategy to dampen destabilizing perturbations. For larger postural challenges, the stiffening strategy alone might be insufficient [5] and a quick hip flexion [6, 7], taking a step or reaching for support might be necessary to recover balance [2]. In young and healthy individuals there is an abundance of physical resources that allow flexibility in the postural control system to manage different postural tasks in different environments with a variety of strategies [2]. Decline in the postural control systems reduces this flexibility, which increases the risk of falling [2]. The natural age-related deterioration of the neuromuscular system is associated with increased reliance on the stiffening strategy for various postural tasks [8–11]. In addition to its relations to physical capacity, the stiffening strategy is correlated with fall-related concerns [12–16], anxiety disorders [11] and decreased executive functioning [8]. Due to its association with aforementioned conditions, stiffening is often described to be a dysfunctional postural control strategy. Proposed negative effects of the stiffening strategy are attenuated net-effect of the agonist [17], increased energy expenditure [18] and poorer physical test performance [10, 16]. Despite its negative associations, agonist-antagonist co-contractions is an important part of our normal postural control. For example, when learning a novel postural task, stiffening the joints is a strategy to allocate the cognitive resources by limiting the degrees of freedom [1]. Other suggested benefits are improved performance and learning during reaching tasks [19], increased proprioceptive input via I-a afferents [17] and its imperative role when performing fast movements [17]. The current knowledge about the stiffening strategy is incomplete and needs to be further studied to discover the nuances of its role and functions in different contexts.

A viable method to study the stiffening strategy is by estimating joint stiffness by calculating the level of agonist-antagonist co-contractions with electromyography (EMG) based co-contraction indexes (CCI) [20]. Two of the most common indexes are suggested by Rudolph et al.: $\left(\frac{Low}{High}\right)\ast \left( Low+ High\right)$ [21] and Falconer and Winter: $\frac{2\ast Low}{Low+ High}$ [22]; where “Low” represents the muscle with the lowest EMG value for that time period. The index by Falconer and Winter is based on the ratio of the two muscles, i.e., if the EMG signal of the two opposing muscles are equal, the CCI-value would be maximized, regardless of whether the magnitudes are high or low. The index by Rudolph accounts both for the similarity and the magnitude of the EMG signals and has therefore been argued to be preferable for estimating joint stiffness [20].

To study the role of the stiffening strategy among people of older age, we sought to investigate: 1) if increased stiffening is associated with postural control performance during an unpredictable surface perturbation task, and 2) which sensory and motor variables explain feed-forward (i.e., pre-perturbation) and feedback (i.e., post-perturbation) stiffening.

## Methods

### Settings

This study is based on the data sampled for the Balancing Human and RoboT (BAHRT) project. A comprehensive test protocol was executed at the Human Health and Performance Lab – Movement Science at Luleå University of Technology.

### Participants

45 community dwelling adults over the age of 70 was recruited via the Swedish Population Register. For inclusion, the participants had to be able to read 100 pt. large block letters (adjusted sight), be able to stand unassisted for at least 30 seconds and be able to understand simple instructions.

### Surface perturbation task

For the surface perturbation task the participants stood on a six degrees of freedom (6dof) platform (CKAS Mechatronics Pty Ltd., Australia), secured by a harness attached to the ceiling and safety straps held in both hands. They were unaware of exactly when and how the platform would move. The task consisted of a single surface perturbation, without any familiarization of the task, where the platform translated anteriorly for 8 cm at a velocity of 10 cm/s, then, tilted 6° anteriorly (toe down) at a velocity of 11°/second and then back to level surface, aiming to mimic a decelerating bus. The participants were given the task to stay balanced in place, i.e., not taking a step or using a gripping strategy via the safety straps. For further analysis, they were assigned to one of two groups, successful or unsuccessful, according to their postural task outcome.

During the perturbation test, EMG was recorded by a Noraxon DTS 16 channels wireless EMG system (Noraxon Inc., USA) with a sample frequency of 3000 Hz. Ag–AgCl Noraxon dual surface electrodes with a fixed 2 cm inter-electrode spacing was attached to tibialis anterior and medial gastrocnemius bilaterally according to the recommendations by SENIAM after the area had been shaved and rubbed with alcohol to decrease impedance [23]. The first processing of the EMG data were done in the Qualisys Track Manager software (Qualisys Inc., Sweden) where the time to muscle onset was determined by visually assessing the raw EMG, marking the first significant EMG burst of tibialis anterior after the perturbation onset. The latter data handling was done in MATLAB where the EMG data was bandpass filtered 20–500 Hz and root mean squared with a 50 ms sliding window. The EMG signals from the perturbation test was normalized to EMG signals from maximum voluntary isometric contraction (MVIC) tests of each muscle. These isometric tests were done in a BIODEX system 3 dynamometer (Biodex Medical Systems, Inc., USA) and is described below, along with the description of the strength tests of the other muscles of the lower extremity. The level of stiffening was represented by the CCI suggested by Rudolph et al. calculated for each sample. Thereafter, the average CCI was calculated 100-0 ms prior the perturbation onset and 0-100 ms after tibialis anterior muscle onset, quantifying the feed-forward (pre-perturbation)- and feedback (post-perturbation) stiffening period, respectively. Finally, the left and right side CCI for both muscles were averaged for each period, respectively.

### Assessment of sensory and motor functions and fall-related concerns

Adjusted binocular vision acuity was tested with the NFD-chart. The participants stood 5 m from the chart, reading aloud the letters at the chart with increasingly smaller font. Test scores ranges between 0.1 and 2.0, where higher score indicate better vision.

The vestibular system was assessed with aid of a pair of Frenzel glasses. The participants would, with a stationary head, perform horizontal and vertical eye movements as well as both active and passive rotations of the neck at different speeds [24]. The presence of nystagmus during any of the tests was considered as a positive finding for vestibular dysfunction on a dichotomous scale.

Pressure sensitivity were assessed with Semmes Weinstein monofilaments that was pressed perpendicular against the lateral malleols with enough force to bend the filament [25]. Different stiffness of the filaments i.e., providing different forces to the skin was used, starting with the lightest touch of 0.4 g of pressure, increasing the stiffness to 2, 4, 10 and 300 g of pressure. Each stiffness was tested three times, the lightest touch the participant could perceive for right and left side, respectively, was noted.

Joint position sense for the knee, ankle and cervical spine was assessed with active joint repositioning test, which has been used in research for various populations with good psychometric properties [26]. The knee and ankle were tested in the Biodex dynamometer. The participants sat blindfolded, with the knees flexed at 90 degrees. They actively extended the knee to 30° where the Biodex stopped the motion and they were asked to memorize the position for 4 seconds. The participants then relaxed and went back to the starting position. Then they were asked to actively reproduce the target position as accurate as possible. The test was repeated three times, reverting to the starting position between each trial. Joint position sense of the ankle was assessed similarly but with starting position at 20° plantar flexion and target position at 5° of dorsal flexion. The absolute error mean of the three trials was calculated for each joint and side.

Joint position sense of the neck was tested with the Qualisys Pro Reflex capture system (Qualisys Inc., Sweden). Four reflective markers were affixed at the head with a headband, positioning two markers at each side of the forehead and two markers at the corresponding site on the back of the head. Six markers were positioned at the trunk (two at sternum, one at each acromion, one at the spinal process of the seventh cervical vertebrae and one at the spinal process of the tenth thoracic vertebrae, making up a rigid body. The participants sat in neutral spinal position, actively rotating the neck approximately 45° to the right side and then trying to actively reposition the neck to the neutral position. This was repeated 6 times per direction, both left and right. The mean absolute error of the rotational angle between the head and the trunk segment were calculated for each side [27].

Non-postural reaction time was tested with participant sitting in front of a personal computer with a software that showed a blank screen. The screen suddenly changed color from black to green and simultaneously gave an audible beep signal, whereupon the participant would hit the space button on the keyboard as fast as possible. The average reaction time of five attempts was calculated.

Maximum isometric leg muscle strength was tested in the Biodex dynamometer. Hip extension and abduction were tested in prone with 90° knee flexion and side lying with a straight knee, respectively. The lever of Biodex was positioned distally at femur, just proximal the condyles. Knee flexion and extension was performed sitting with 30° knee flexion and the lever positioned distally at the lower leg, just proximal to the malleoli. Both ankle plantar- and dorsal flexion torque was tested sitting with the seat tilted back 55°, the femurs supported and the lower legs parallel to the floor. This reclined position created a slight angle at the knees and a neutral position of the ankles. The feet were strapped to a pedal to test both plantar- and dorsal flexion torque. While testing, MVIC EMG of the tibialis anterior and gastrocnemius was sampled for the EMG normalization procedure described above. Each muscle strength test was repeated three times for 3 seconds under strong encouragement from the test leader. The maximum achieved torque for each test was normalized to the height of respective participant.

Fall-related concern was measured with Fall Efficacy Scale-International (FES-I) [28].

### Statistical analysis

To evaluate relations between task failure and the level of stiffening within each period, a binary logistic regression was executed using IBM SPSS Statistics for Windows, Version 26 (IBM Corp., Armonk, NY,. USA). The feed-forward and feedback CCI was, due to skewness, Log_10_ transformed. Thereafter, the CCI-values were standardized to use the generated Z-values as independent variables. The dichotomous variable, whether the participant successfully managed the platform perturbation or not was set as the dependent variable. The level of significance was set at 0.05. Group difference of the descriptive data between the successful and unsuccessful group were tested with Mann-Whitney U test for continuous variables and Fishers exact test for dichotomous variables using SPSS. The variables attained from the sensory and motor test protocol were imported to SIMCA 15 (Sartorius Stedim Data Analytics AB, Umeå, Sweden) where two separate Orthogonal Projections to Latent Structures (OPLS)-models was generated, one with the CCI for the feed-forward- and one with CCI for feedback period as dependent variables.

As the applied CCI by Rudolph et al. is responsive to the amplitude of the normalized EMG signal i.e., if all else being equal, weaker individuals will show a higher CCI as they use more of their muscular capacity to produce a certain joint torque. Therefor a post-hoc OPLS-model was generated to control the impact of strength on feedback stiffening. The same independent variables were used as for the original OPLS-feedback model, but with the CCI by Falconer and Winter as dependent variable.

## Results

### Missing data

Four of the participants did not perform the surface perturbation test as it was considered too challenging by the participant or the test leader. The data of additionally seven participants could not be included due to serious signal disturbances (*n* = 6) and data file corruption (*n* = 1). The final sample consisted of 34 individuals; descriptive data are presented in Table 1. Only one participant had positive signs for nystagmus, hence this variable was excluded from the analysis.

**Table 1:** Descriptive data of the groups “Successful” and “Unsuccessful”

Characteristics	Successful (*n*=13)	Unsuccessful (*n*=21)	Sig.
Subject characteristics			
Age (Years)	74 (72 – 75)	75 (71 – 77)	0.441^a^
Sex (Male/Female)	8/5	6/15	0.080^b^
Height (cm)	170 (161 – 174)	166 (161 – 170)	0.362^a^
Weight (Kgs)	72 (65 – 81.5)	74.5 (57 – 80)	0.576^a^
FES–I	18 (17–23)	19 (17–24)	0.462^a^
Sensory testing			
Visual Acuity	0.8 (0.8–0.9)	0.7 (0.7–0.9)	0.292^a^
Pressure Sense Left (Monofilament thickness, mm)	2 (2–4)	2 (2–4)	0.807^a^
Pressure Sense Right (Monofilament thickness, mm)	2 (0.4–4)	2 (2–4)	0.701^a^
JPS Neck Left (Mean error, Degrees)	2.1 (1.0–4.1)	4.0 (1.9–6.0)	0.205^a^
JPS Neck Right (Mean error, Degrees)	2.4 (1.6–4.2)	4.1 (2.1–6.7)	0.169^a^
JPS Knee Left (Mean error, Degrees)	4.3 (3.3–4.7)	4 (3.3–5.3)	0.807^a^
JPS Knee Right (Mean error, Degrees)	4 (3–5.7)	3.7 (3.2–5.1)	0.957^a^
JPS Ankle Left (Mean error, Degrees)	3 (2.7–4.7)	4.7 (3.7–5.4)	0.221^a^
JPS Ankle Right (Mean error, Degrees)	3.7 (1.7–4.3)	3.7 (3.2–6.2)	0.316^a^
Muscle strength testing			
Hip Extension Left (Nm)	50.4 (35.3–67.4)	46.2 (35.7–54.6)	0.400^a^
Hip Extension Right (Nm)	52.6 (34.6–79.5)	46.2 (39.2–57.9)	0.246^a^
Hip Abduction Left (Nm)	55.7 (33.5–76.2)	46.9 (27.8–65)	0.344^a^
Hip Abduction Right (Nm)	54 (34.8–75.1)	54.4 (39.9–69.69	0.917^a^
Knee Extension Left (Nm)	92.2 (68.5–112.3)	73.1 (61–95.7)	0.205^a^
Knee Extension Right (Nm)	79.5 (68.5–112.7)	76.2 (61.7–97.1)	0.701^a^
Knee Flexion Left (Nm)	74.4 (58.6–91.1)	54.6 (50.9–79.7)	0.129^a^
Knee Flexion Right (Nm)	86.8 (57.2–92.2)	56.1 (48.7–84.8)	0.158^a^
Ankle Dorsal flexion Left (Nm)	20.7 (19–30.6)	19.7 (17–23.2)	0.148^a^
Ankle Dorsal flexion Right (Nm)	20.1 (17.6–37.4)	24.3 (17.9–25.8)	0.701^a^
Ankle Plantar flexion Left (Nm)	91.4 (59.7–130.7)	72.4 (64.5–95.3)	0.362^a^
Ankle Plantar flexion Right (Nm)	90.7 (55.1–107.7)	72.4 (57.5–102.4)	0.552^a^
Non-postural computer reaction test			
Non-postural reaction time (ms)	354 (326–367)	390 (358–439)	0.082^a^
Surface perturbation task			
Time to muscle onset	160 (140–253)	155 (135–261)	0.899^a^
CCI Feed–forward	0.02 (0.01–0.04)	0.02 (0.02–0.03)	0.344^a^
CCI Feedback	0.08 (0.06–0.14)	0.16 (0.09–0.29)	0.046^a^

Characteristics are presented as median and interquartile range. a Mann-Whitney U test; b Fishers Exact test. Bold text indicate significant group difference. Strength variables are presented as raw value, Newton-metre (Nm). Abbreviations: JPS Joint Position Sense, FES-I Falls Efficacy Scale – International, CCI Co-contraction Index.

### Postural control success

**Fig. 1:**
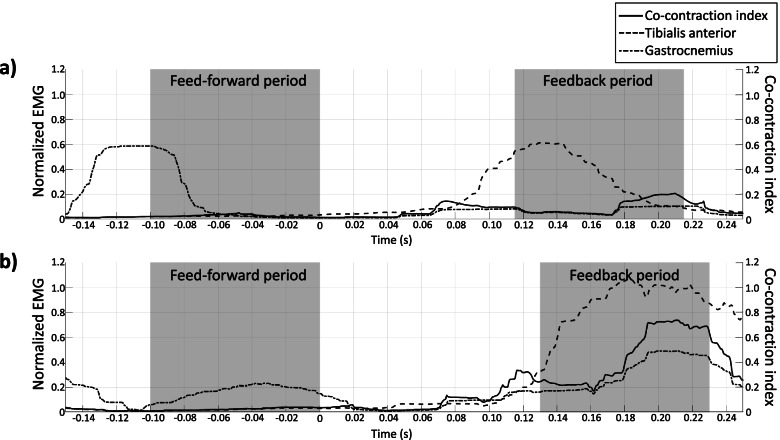
Representative EMG and CCI from a successful and unsuccessful participant. The normalized EMG for the right tibialis anterior and gastrocnemius as well as the CCI of the two muscles during the feed-forward- and feedback period. **a**) Depicts the result from a participant who was successful in the postural task. **b**) Shows a participant who was unsuccessful in the postural task

**Fig. 2:**
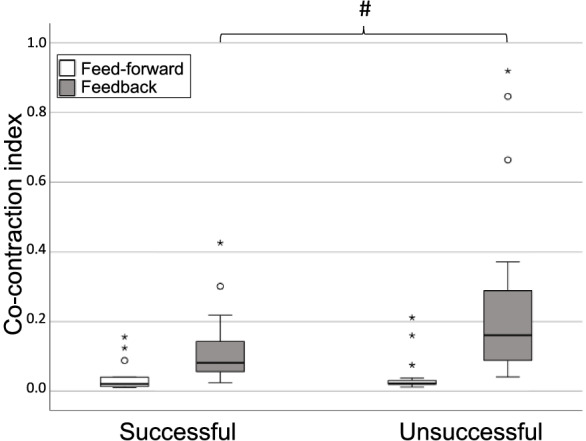
CCI-Box plots for the groups with successful and unsuccessful postural control outcome. The white and the gray boxes represent the CCI-values for the feed-forward and feedback period, respectively. The two leftmost boxes represent the CCI-values for the successful group for each period, the two rightmost boxes represent the CCI-values for the unsuccessful group. The boxes contain the interquartile range (IQR), the medians are marked with a bold line, the T-bars contain max and min CCI-values, outliers excluded.  ○ = CCI between 1.5–3 times the IQR; ★ = CCI above 3 times the IQR; # = Significant group difference, tested with Mann Whitney U test

The binary logistical regression analysis showed that higher levels of feedback- (*P* = 0.034; OR 2.880; 95% CI 1.083–7.657) but not feed-forward stiffening (*P* = 0.478; OR 0.740; 95% CI 0.322–1.701) was significantly correlated with poorer postural control success after an unexpected surface perturbation task. The regression model showed a Negelkerke R^2^ of 0.212 with a predictive value of 76.5%.The normalized EMG and CCI from one participant representing each group are shown in Fig. 1. The dispersions of CCI-value for the feed-forward and feedback periods for the successful and unsuccessful group are depicted in Fig. 2.

### Feed-forward control

Feed-forward stiffening was not explained by the independent variables as the OPLS model for the feed-forward sequence came out poor, with an explained variance (R^2^Y) of 10.9% and a predictive value (Q^2^) of − 7.3%. Permutations of the model shows an invalid model, see Additional file 1 Fig. A1.

### Feedback control

**Fig. 3:**
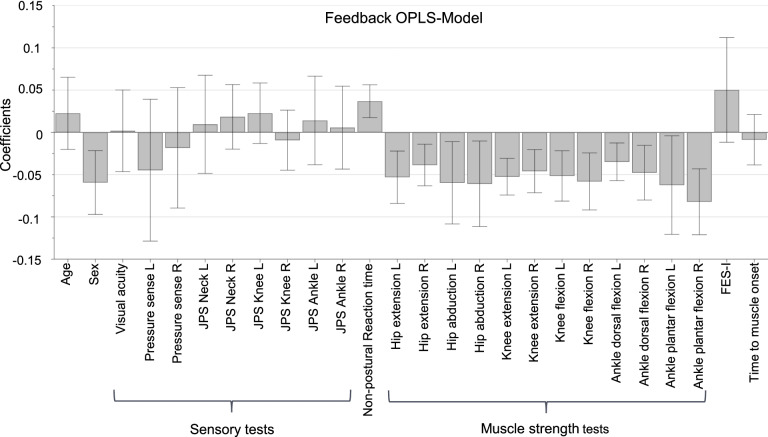
Coefficients for the feedback OPLS-model. The direction of the bars shows positive or negative associations between variables and increased ankle stiffening as a feedback response to the perturbation. The error bars not including zero indicate that the variable is significant in the model. L = Left side; R = Right side; JPS = Joint Position Sense; FES-I = Falls Efficacy Scale – International

The OPLS-model with feedback CCI as dependent variable explained 40.6% (R^2^Y) of the variance and had a predictive value (Q^2^) of 32.2%. Figure 3 shows that having slow non-postural reaction time, poor lower extremity strength in all tested muscles and being female, are correlated with higher levels of feedback stiffening. A permutation plot found in Additional file 1, Fig. A2 shows a valid model.

## Discussion

The results showed that a higher level of feedback stiffening was associated with poorer postural control performance, which was explained by having slow non-postural reaction time, poorer leg muscle strength and female sex. Feed-forward stiffening was not correlated with postural task success and was poorly explained by the variables investigated in this study.

The binary logistic regression shows that individuals with one standard deviation higher CCI-value during the feedback period, have about a threefold higher risk of failing the surface perturbation task. This might be explained by an attenuated agonist torque and/or that a rigid body will inherently be less stable than a more compliant body [17]. Due to its correlations with poor postural control outcome, high levels of feedback stiffening could be considered an indicator to poorer postural control and possibly a proxy for and increased risk of falling. Regarding the feed-forward period, the non-significant negative association with task failure and stiffening suggests that it does not have a negative effect on dynamic postural control, maybe due to an increased muscle excitability [17]. However, we measured feed-forward stiffening during a task where the participant did not know what to expect, a more predictable task might show other relations with feed-forward stiffening and postural control [29].

Leg muscle strength proved to be important to explain feedback stiffening, which was confirmed by the post-hoc analysis with the CCI by Falconer and Winter (Additional file 1, Fig. A3). Stronger legs could indicate higher levels of physical activity [30]. But physical activity alone has been reported to be poorly associated with balance performance [31, 32], which supports the independent importance for reduced strength itself as a physical motive for using the stiffening strategy as a response to unpredicted surface perturbations. As leg muscle strength explains feedback stiffening, which correlates with poorer postural control success, the results could just show that weaker individuals are less likely to manage postural perturbations with a feet-in-place strategy. It is however possible that the stiffening strategy is an adequate postural control strategy for weaker individuals in some situations. But in more challenging tasks, they are due to their lower physical capacity unable to generate a matching response to the postural perturbation.

We found that slower non-postural reaction time correlates with higher CCI-values in the feedback OPLS-model. However, no such correlation was found between time to muscle onset during the perturbation and feedback stiffening. This discrepancy might be due to that postural reaction time are dependent on the monosynaptic stretch reflex, relaying at the spinal level. Whereas the non-postural reaction time are processed on a cortical level. According to the processing speed theory, cortical processing speed are important for cognitive functions [33], and reaction time tests are valuable instruments to estimate cognitive function [34]. Hence, we suggest that non-postural reaction time might serve as a proxy for cognitive function, which has been associated with the level of stiffening during walking [8] and surface perturbations [35]. However, the cognitive capacity and its relations to stiffening during the perturbation task was not in the scope for this study and needs to be investigated in future studies.

Sex proved to be an important factor for the feedback period where women display higher CCI-values than men. Due to the small sample size, we were not able to make separate analyses of men and women to find specific factors for each sex that explain the stiffening strategy. The literature shows that women have higher risk of falling compared to men [36–38]. This difference is multifaceted where, among other factors, women have lower muscle mass [39] and suffer more from fall-related concerns [40], which in previous research have been associated with the stiffening strategy [12–16] and falls [5, 41]. We did not find a correlation with the FES-I and stiffening in our unpredicted surface perturbation test.

The sensory systems showed no significant importance for the OPLS-models. The results suggest that, in a population of community dwelling older adults, the stiffening strategy during unpredictable surface perturbations are neither the result from- nor compensating for sensory deficits.

Visual acuity showed a very small and non-significant impact on both models. Vision has undoubtedly a crucial role in postural control and visual impairment have shown to be associated with a history of falling [42], probably due to not detecting obstacles or unevenness in the environment. The sight is a relatively slow sensory system with primarily anticipatory qualities and thus has an inferior role in managing unexpected surface perturbations [43].

Only one participant showed signs of vestibular dysfunction which is in sharp contrast with the literature that suggests approximately one third of the American population over the age of 40 suffers from vestibular dysfunction [44]. Hence, the vestibular variable was excluded from further analysis.

Although poorer pressures sense, examined with Semmes-Weinstein monofilament, has been associated with poorer physical performance and fall prediction [45, 46], our model did not show an association between pressure sense and the stiffening strategy during an unpredicted postural perturbation.

Joint position sense at the neck, knees and ankles showed to have little impact on the stiffening strategy. This is in contrast with a review by Henry and Baudry that concluded that age-related deterioration of leg proprioception increases co-contraction levels [9]. Compared to the protocol of this study, the reviewed studies used simpler and more predictable postural tasks; such as quiet stance [10, 47–49] and uphill walking [50]. This shows how the relations between postural control and the sensory and motor variables might vary in different contexts.

People who are at higher risk of falling are more prone to use a stepping strategy to manage a postural threat, and might do so effectively in everyday situations [51]. In this protocol the participants were encouraged to use a feet-in-place strategy. Consequently, some participants were forced to use a strategy that they wouldn’t have used in a real-life situation. As stiffening is associated with unfamiliar tasks [1], this forced task execution might have led to higher CCI-values for those that would rather use a stepping strategy. However, this methodology is accepted in clinical and laboratory studies to standardize the task [51].

We chose to use OPLS-modeling as it allow analyzing noisy data with relatively many independent variables in relation to the sample size [52].

### Limitations

With this cross-sectional study, we found some interesting correlations that warrant longitudinal studies to investigate causality. Regarding the protocol, there are some limitations to acknowledge. Firstly, a more sensitive test of the vestibular system would have allowed a fairer estimation of vestibular function in the sample. Secondly, it would have been valuable to include a thorough assessment of cognitive functions. This would have validated our hypothesis that, based on the reaction time test, cognitive function play a major part in feedback stiffening. Thirdly, perhaps a direct rating of the perceived fear of falling during the surface perturbation task, instead of the FES-I, would have depicted a fairer estimation of context dependent fall-related concerns. Fourthly, a larger sample would have allowed for some additional analyses, e.g., separate OPLS-models to see if the sensory and motor systems have different influences on the stiffening strategy between men and women.

### Future directions

To further explore the stiffening strategy, future studies should investigate if interventions targeted at decreasing the level of feedback stiffening will lead to more successful postural control during unpredicted surface perturbations. In relation, it would be interesting to see if interventions targeting leg muscle strength and/or reaction time will automatically decrease stiffening. In this study, we examined the stiffening strategy in relation to anticipatory and reactive actions to an unexpected experimental task. We warrant research investigating both predictable and unpredictable postural tasks, as context might impact the relations of sensory and motor variables and postural control. Our results also support further investigation of the stiffening strategy’s relations to sex and/or gender as well as psychological and cognitive factors.

## Conclusions

Higher level of feedback stiffening was associated with poorer postural control performance during an unpredicted surface perturbation task, which was explained by slower non-postural reaction time, poor leg muscle strength and female sex; but not the acuity of the sensory systems. It remains to be answered if feedback stiffening is the best available strategy for individuals with these traits, but due to their poorer physical capacity, they are unable to successfully respond to an unpredicted surface perturbation. Feed-forward stiffening showed poor relation to postural control efficiency and was poorly explained by the sensory and motor variables. To reveal the causal relationship between the stiffening strategy and postural control outcome, future studies should investigate if targeted interventions affect the levels of stiffening and thereby postural control success.

## Supplementary Information

**Additional file 1.**

## Data Availability

The datasets used and/or analyzed during the current study are available from the corresponding author on reasonable request.

## Abbreviations

CCI: Co-contraction index
OPLS: Orthogonal Projections to Latent Structures OR Orthogonal Partial Least Squares
IQR: Inter quartile range
FES-I: Falls Efficacy Scale – International
