# Supplementary material for: Increased co-contraction reaction during a surface perturbation is associated with unsuccessful postural control among older adults
Source: BMC Geriatr. 2022 May 19;22:438. doi: 10.1186/s12877-022-03123-2 (PMC9118814; doi:10.1186/s12877-022-03123-2)
Supplement: Supplementary file 1 — Additional file 1. [file 12877_2022_3123_MOESM1_ESM.docx]

**Appendix**


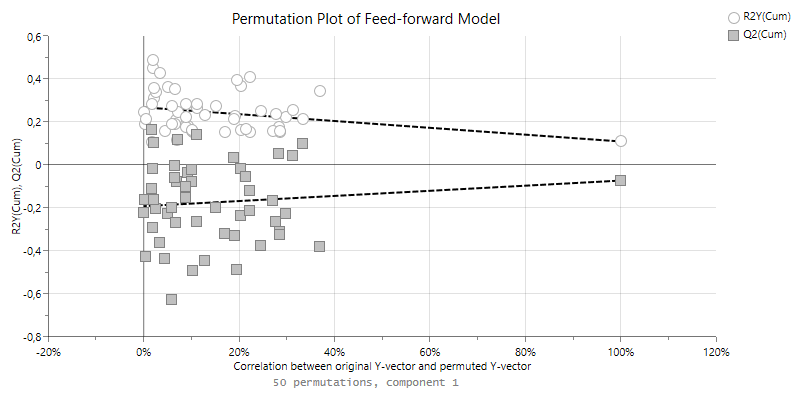


**Figure A1.** Permutation plot of Feedforward model. The circles and squares to the right show the R^2^Y and Q^2^ values of the generated OPLS-model, respectively. The plots to the left show the corresponding values for 50 models where the Y-values are permuted. A regression line is drawn from the true R^2^Y and Q^2^ values trough the cluster of permuted models. This gives an indication of the robustness of the model i.e., how likely the model is to predict the dependent variable for new observations. A strong model has higher R^2^Y and Q^2^ values compare to the permuted models and/or a Q^2^ regression line that intercepts the vertical axis below zero. Hence, the Feedforward model show a very weak model.


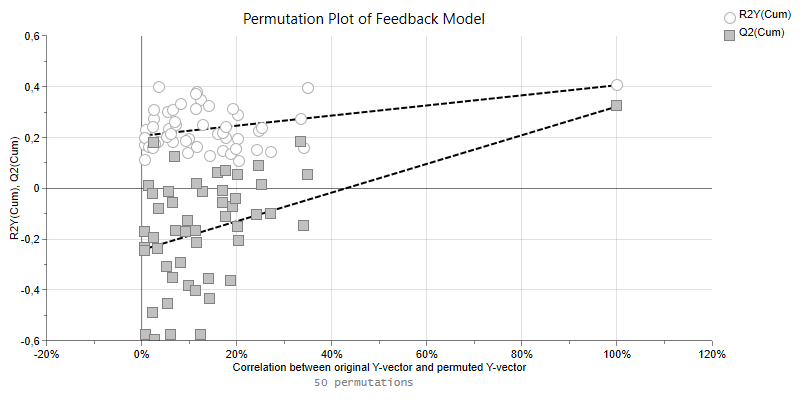


**Figure A2.** Permutation plot of Feedback model. The circles and squares to the right show the R^2^Y and Q^2^ values of the generated OPLS-model, respectively. The plots to the left show the corresponding values for 50 models where the Y-values are permuted. A regression line is drawn from the true R^2^Y and Q^2^ values trough the cluster of permuted models. This gives an indication of the robustness of the model i.e., how likely the model is to predict the dependent variable for new observations. A strong model has higher R^2^Y and Q^2^ values compare to the permuted models and/or a Q^2^ regression line that intercepts the vertical axis below zero. Hence, the Feedback model show an acceptable model.


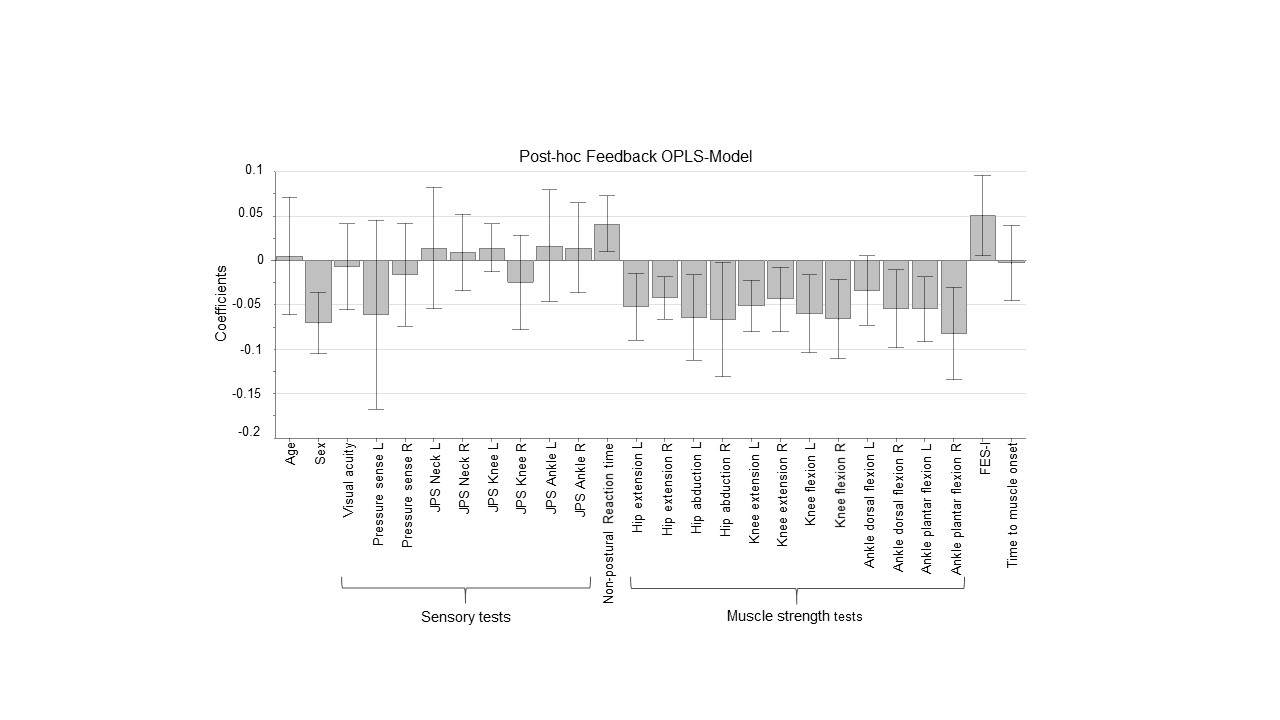


**Figure A3.** Coefficients of Post-hoc Feedback OPLS-Model with the same independent variables as for the original feedback model, presented in Figure 2, but with the CCI suggested by Falconer and Winter [20]. This model was generated to control the importance of lower extremity strength as the different CCIs have different qualities. This model shows a similar result as the original model except that Left ankle dorsal flexion strength is not significant and that the variable FES-I is significantly important for the model to explain feedback stiffening. The model has an explained variance (R^2^Y) of 43% and a predictive value (Q^2^) of 33%. This model confirms that strength is an important variable to explain feedback stiffening due to an unpredicted surface perturbation.
